# Survival rate of dental implants installed by postgraduate students attending an implantology program in Brazil: A 52-month retrospective analysis

**DOI:** 10.3389/fdmed.2023.1170253

**Published:** 2023-04-18

**Authors:** Myungjin Kang, Henrique Smanio Neto, André Antonio Pelegrine, Cecilia Pedroso Turssi, Juliana Trindade Clemente-Napimoga, Marcelo Henrique Napimoga

**Affiliations:** ^1^ Faculdade São Leopoldo Mandic, Instituto São Leopoldo Mandic, Implantology, Campinas, Brazil; ^2^ Faculdade São Leopoldo Mandic, Instituto São Leopoldo Mandic, Restorative Dentistry, Campinas, Brazil; ^3^ Faculdade São Leopoldo Mandic, Instituto São Leopoldo Mandic, Laboratory of Neuro-Immune Interface of Pain Research, Campinas, Brazil

**Keywords:** implantology, dentistry, dental implant, implant survival rate, education

## Abstract

**Objectives:**

The aim of this study was to assess the survival rate and identify possible risk factors for failure of dental implants placed by postgraduate students in Implantology at a Brazilian Dental School.

**Materials & methods:**

A retrospective observational study was conducted to evaluate 1,164 dental implants placed by postgraduate students in Implantology at São Leopoldo Mandic Dental School (Brazil) during a 3-year time period (2018–2020). Data collected from the patients' medical charts included the following: implant loss, gender, diabetes, smoking, continuous use of medication, type of implant connection system, implant position (maxilla or mandible), previous bone grafting and type of prosthetic provisioning (temporary prosthesis, immediate prosthesis or permanent prosthesis). The association between all the independent variables and implant loss was run using *χ*^2^ and *G* tests (*α* = 5%). The implant survival rate was estimated using Kaplan-Meier curve.

**Results:**

Gender, diabetes, smoking, continuous use of medication, type of implant connection system, implant position, previous bone grafting and type of prosthetic provisioning showed no statistically significant association with implant loss. Of the 1,164 implants installed, 29 (2.5%) failed. The overall survival rate of dental implants placed by postgraduate students up to 52 months was 90.5% (IC95%: 74.5%–96.7%).

**Conclusions:**

Implants placed by postgraduate students in Implantology at São Leopoldo Mandic Dental School showed a high survival rate, with gender, diabetes, smoking, continuous use of medication, type of implant connection system, implant position, previous bone grafting and type of prosthetic provisioning not accounting for the risk of implant failure.

## Introduction

The use of dental implants is considered one of the most prominent scientific breakthroughs and predictable treatment options to restore partially and totally edentulous patients. Thus, the large-scale use of dental implants, which demonstrate predictable long-term results from a functional, aesthetic and peri-implant health point of view, has high survival rates well demonstrated in the literature (1). Recent studies reported 86%–98% survival rates for dental implants after 5 years of follow-up (2, 3) and around 90% even after 10 years of follow-up (4, 5).

Implant-related complications have been categorized into two main types: biological and technical. Among the biological complications, some of the patient-related risk factors include smoking and systemic diseases such as uncontrolled diabetes mellitus (DM); and periodontitis; which are all characterized as patient-related risk factors for implant failure (6, 7). From a technical point of view, clinical training in implant dentistry provides graduate dental students with advanced skills. Although many studies report the success of implant rehabilitations, there is limited literature on the survival of implants performed by postgraduate students. A recent study evaluated the survival rates of implants and prostheses placed by undergraduate students in a dental hospital. The study was a retrospective university/hospital based study and included patients visiting the dental hospital. Of the 86,000 patients who visited Saveetha Dental College, a total of 79 patients were enrolled in the study according to the inclusion criteria of patients who had undergone implant surgery by undergraduate students. The survival rate from implants placed was 92.4% (8). Another study based in the rehabilitation of patients with implants at the University of Alberta (Canada) by undergraduate students, evaluated 289 implants in 189 patients, with only 1 loss. Therefore, a high survival rate of 99.7% was verified (9).

It is well known that the use of different dental implant brands can result in different clinical outcomes (10). However, a number of other local and systemic conditions may also impair the implant therapy (11). Therefore, the use of a single dental implant brand in an investigation seems to be reasonable to identify possible risk factors for failure of dental implants other than the implant brand itself. In this scope, a recent retrospective study showed, by evaluating 6,113 implants, that also gender and previous bone augmentation history at the implant site must be considered risk factors for early implant failure (12).

Hence, the aim of this single-center, retrospective study was to assess the survival rate and identify possible risk factors for failure of dental implants of the same brand, placed by postgraduate students in Implantology at a Brazilian Dental School. We tested the null hypothesis that dental implants placed by postgraduate students in Implantology would not fail influenced by patients' gender, habits and health condition, bone grafting, implants' design and position, and type of prosthetic provisioning.

## Material and methods

### Ethics

The research project was approved by the Research Ethics Committee of Faculdade São Leopoldo Mandic, registration number # 49980221.7.0000.5374. All patients included in this study provided written informed consent prior to implant treatment as well as the patients under the age of 18 years the consent was obtained from parents or guardians of the minors. All personal patient data were automatically fully anonymized by the institutional program for medical records before the researchers accessed the medical records.

### Study subjects

The present study is a single-arm, retrospective observational study based on the dental implants placed by postgraduate students in Implantology at São Leopoldo Mandic (Brazil) during a 3-year time period (2018–2020), based on the data available in the medical records of patients. This observational study was conducted according to the guidelines of Strengthening the reporting of observational studies in epidemiology (STROBE).

### Non-inclusion, inclusion and exclusion criteria

Non-inclusion criteria were any systemic condition, except for diabetes, alcoholism and dependence on drugs. Eligible for the study were patients between 17 years and 10 months and 82 years of age whose records indicated that they received Intraoss dental implant system between January 1, 2018, and December 31, 2020. The patient who received the dental implant at the age of 17 years and 10 months of age had reached skeletal maturity confirmed by x-ray. Just patients that received implants made by the Intraoss brand (Itaquaquecetuba, SP, Brazil) were selected as they account for 40.5% of all placed implants, while the remaining 59.5% pertained to 5 other brands [Conexão Sistemas de Próteses (Brazil), Sin Implant System (Brazil), Nobel Biocare (Switzerland), Straumann (Switzerland) and Implacil (Brazil)].

The exclusion criteria were patients whose records indicated that they received another dental implant system during the analyzed period.

### Data collection and analyses

Data collected from the patients' medical charts were submitted to descriptive and inferential analyses, using *χ*^2^ and *G* tests, to investigate the association between implant loss and gender, diabetes, smoking, continuous use of medication, type of implant connection system, implant site (maxilla or mandible), previous bone grafting and type of prosthetic provisioning (temporary prosthesis, immediate prosthesis or permanent prosthesis). The implant survival rate was estimated using the Kaplan-Meier curve. The level of significance was set at 5% and statistical calculations were performed using SPSS 23 (SPSS INC., Chicago, IL, USA) and BioEstat 5.0 (Fundação Mamirauá, Belém, PA, Brazil).

## Results

During the 2018–2020 period, postgraduate students in Implantology at São Leopoldo Mandic performed a total of 2,875 implants. According to the inclusion criteria, a total of 1,164 dental implants, which had been installed in the oral cavity of 742 patients were included in the study. Of the total number of patients included in this study 254 (34.2%) were men and 486 (65.5%) were women. For two (0.3%) patients, information regarding gender was non-existent. The age of the patients ranged from 17 to 82 years old (average: 55.1 ± 11.5 years). Only one of the 742 patients had no information regarding age.

Fifty-four (7.3%) out of the 742 patients had diabetes, with 26 (3.5%) being men and 28 (3.8%) women, while 680 (91.6%) had no diabetes and for 8 patients this information was unavailable.

Smoking was identified in 96 (12.9%) of the 742 patients, of whom 40 (5.4%) were men and 56 (7.5%) were women. Non-smokers summed 643 (86.7%) patients and three others had no information about smoking. Sixteen (2.2%) patients were both smokers and diabetics.

Of the 742 patients, 354 (47.7%) were on continuous medication, of which 109 (31.9%) were men, 244 (32.9%) were women and one did not report their gender. Non-users of medication totaled 385 (51.9%) patients and other three did not have information on this aspect.

In the oral cavity of the 742 patients, 1,164 implants were installed, indicating an average of 1.6 implants per patient. The maximum number was six implants in the same patient. Of the 1,164 implants installed, 907 (77.9%) were tapered connection system, 174 (14.9%) were external connection and 80 (6.9%) were internal connection. For three implants the system connection was unknown.

Of the 1,164 implants, 605 (52.0%) were installed in the maxilla, 530 (45.5%) in the mandible, and for 27 of them the location was not indicated. Bone grafting procedures preceded the installation of 278 (23.9%) of the 1,164 implants, while for the remaining 886 there was no grafting or this information was non-existent (for three implants).

**Figure 1:**
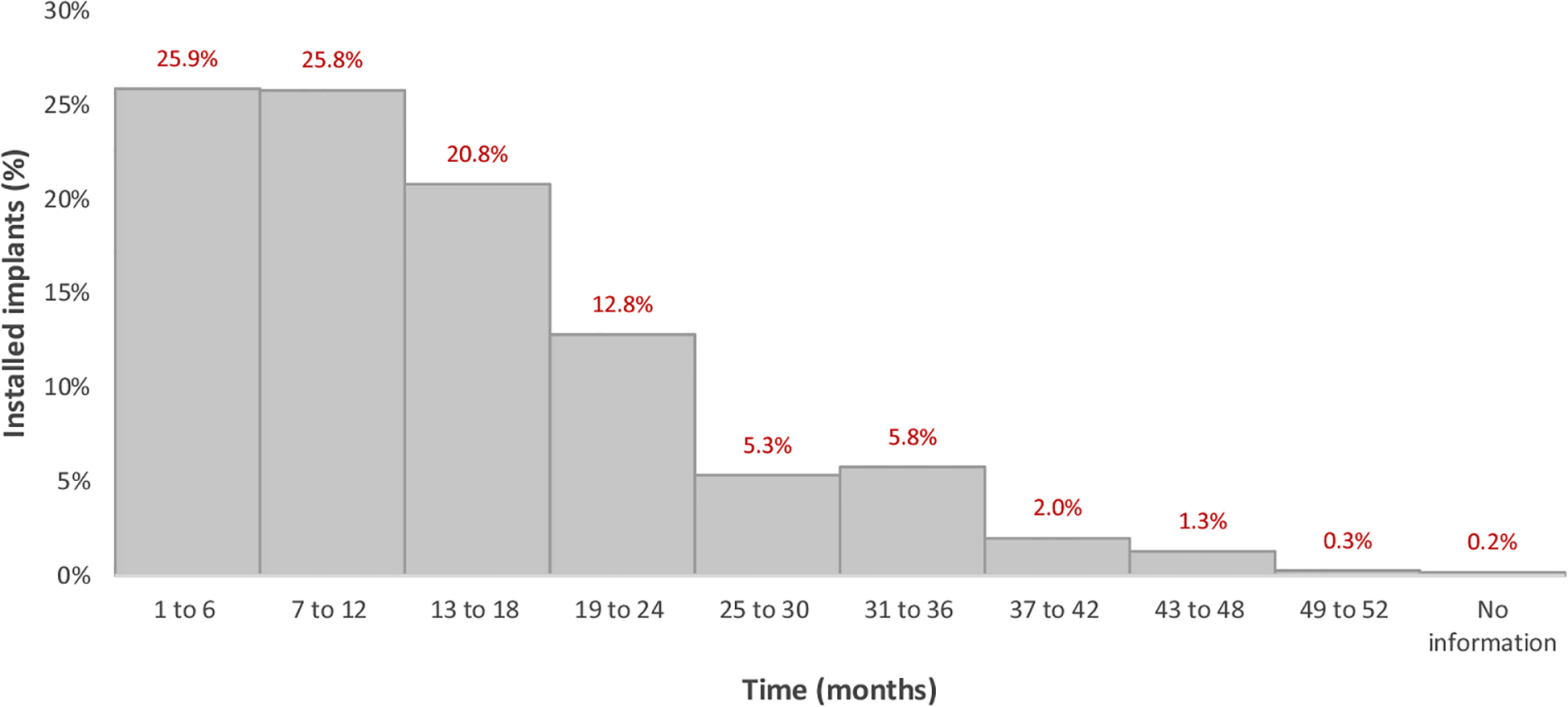
Histogram of the installation time of the evaluated implants.

The mean time of implant installation prior to the data collection was 14.2 months (±10.2 months), with the shortest time being one month and the longest 52 months. Figure 1 is a histogram showing the number of implants that had been placed within each 6-month period. More than half (601) of the 1,164 implants, corresponding to 51.6%, had been installed up to 12 months prior to the data collection, while 992 (85.2%) had been installed up to 24 months previously. For two implants, there was no information on the installation time.

Among the 1,164 implants, 385 (33.1%) received a temporary prosthesis, 257 (22.1%) received an immediate prosthesis and 364 (31.3%) received a permanent prosthesis. Provisionalization information was absent for 158 implants.

Of the 1,164 implants installed, 29 (2.5%) failed. Investigating the association of implant losses with valid responses (excluding cases with no information) there was no statistically significant association with gender, diabetes, smoking, continuous use of medication, type of connection system, implant placement site, previous bone grafting and type of prosthetic provisioning (Table 1).

**Table 1:** Absolute and relative frequencies (%) of implant loss according to sex, diabetes, smoking, continuous use of medications, type of implant system, location of installation, previous bone grafting and type of prosthetic provisioning.

Independent Variable	Implant loss		*p*-value
	Y	N	
**Gender**			
Male	11 (2.7%)	398 (97.3%)	0.755^*^
Female	18 (2.4%)	735 (97.6%)	
	29	1.133	
**Diabetes**			
Y	2 (2.5%)	78 (97.5%)	0.966^**^
N	26 (2.4%)	1.047 (97.6%)	
	28	1.125	
**Smoking**			
Y	6 (3.8%)	154 (96.2%)	0.274^*^
N	23 (2.3%)	978 (97.7%)	
	29	1.132	
**Continuous use of medications**			
Y	12 (2.1%)	555 (97.9%)	0.416^*^
N	17 (2.9%)	577 (97.1%)	
	29	1.132	
**Implant connection system**			
Tapered connection	21 (2.3%)	886 (97.7%)	0.704^**^
External connection	5 (2.9%)	169 (97.1%)	
Internal connection	1 (1.3%)	79 (98.7%)	
	27	1.134	
**Implant placement site**			
Maxila	11 (1.8%)	594 (98.2%)	0.132^*^
Mandible	17 (3.2%)	513 (96.8%)	
	28	1.107	
**Previous bone grafting**			
Y	7 (2.5%)	271 (97.5%)	0.895^*^
N	21 (2.4%)	862 (97.6%)	
	28	1.133	
**Prosthesis**			
Temporary prosthesis	8 (2.1%)	377 (97.9%)	0.543^**^
Immediate prosthesis	5 (1.9%)	252 (98.1%)	
Definitive prosthesis	10 (2.7%)	354 (97.3%)	
	23	983	

For each independent variable, cases without information were disregarded for applying the analyses.

^*^ *p*-value of the *χ*^2^ test.

^**^ *p*-value referring to the *G* test.

**Figure 2:**
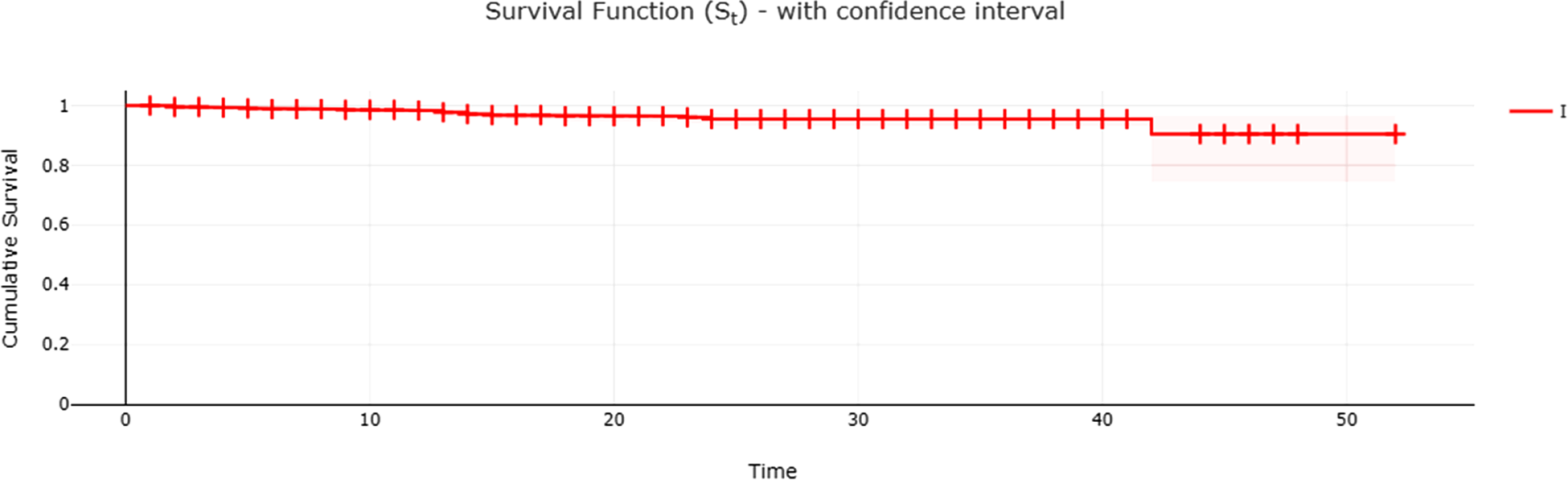
Kaplan–Meier curve for the event of implant loss in the sample evaluated.

Among the 29 implants that failed, one had no information about the placement time. The same occurred for an implant that did not fail. Therefore, of the 1,164 placed implants, 1,162 were considered for estimating the survival rate. Figure 2 shows the Kaplan-Meier curve, with a 95% confidence interval up to 52 months after implant placement, and reveals that the overall survival rate was 90.5%. Table 2 indicates the survival rates in the other time intervals and presents the confidence intervals (95%). The raw data are available as Supplementary File S1.

**Table 2:** Survival rate and confidence interval (95% CI) according to implant placement period.

Time (months)	Survival rate	CI (95%)
1–6	98.84%	97.97% a 99.34%
7–12	98.27%	97.15% a 98.95%
13–18	96.46%	94.68% a 97.66%
19–24	95.51%	93.08% a 97.10%
25–30	95.51%	93.08% a 97.10%
31–36	95.51%	93.08% a 97.10%
37–42	90.48%	74.51% a 96.66%
43–48	90.48%	74.51% a 96.66%
49–52	90.48%	74.51% a 96.66%

## Discussion

Some individual biological factors are known to potentially impair implant prognosis. A meta-analysis based on implant and patient-related data showed a significant increase in the relative risk of implant failure in patients who smoked >20 cigarettes per day compared with non-smokers (13). In the present study we did not observe a positive association of smoking and dental implant failure, however, we did not have the information on how many cigarettes were used per day by each patient, which may bias the results. Importantly, it cannot be ruled out that the lack of association between smoking and failure can be partly attributed to the time since implant placement in our study was up to 52 months. In addition, when working with a dichotomous assessment (yes vs. no for smoking) it is difficult to show effects that are known to be more expressive, thus, if number of cigarettes/packs categorizes it may favor finding an association.

The long-term hyperglycemia of diabetes usually leads to failure, damage, and/or dysfunction of many tissues and organs mainly due to the correlation between glycemic control and the development of microvascular and macro-vascular complications (14). Previous results already demonstrated that diabetic patients presented a statistically significant higher risk of dental implant failure and higher marginal bone loss than non-diabetic patients, mainly type 1 diabetes (15). Our results have not demonstrated statistically significant association between diabetes and dental implant failure. Probably, one explanation for this result might be that patients had diabetes under control, which can prevent peri-implant bone loss (16).

With regard to diabetes, however, one should bear in mind that the data on the presence or absence of diabetes were based only on patient self-report, which requires some caution in data analysis, as no laboratory tests were performed. Nevertheless, it is worth noting that another study verified no association between implant loss and different variables such as bone augmentation, time of implant placement, diabetes and smoking, corroborating our results (17). Besides, in a previous study several parameters similar to those evaluated herein did not yield any significant association with implant failures (18). Therefore, the null hypothesis that dental implants placed by postgraduate students in Implantology would not fail influenced by patients' gender, habits and health condition, bone grafting, implants' design and position, and type of prosthetic provisioning was accepted.

Concerning the type of implant connection, a recent study (19) showed no significant difference in the cumulative survival rate between implants with external and internal abutment connections. These results seem to corroborate the findings of the present study. Moreover, regarding the type of prosthesis used over these implants, it is important to state that, although the prosthetic types were divided into 3 groups in this study (i.e., temporary prosthesis, immediate prosthesis and permanent prosthesis), the situation for each prosthetic type varied more than could be captured by a tripartite classification (e.g., number of splinted units and size of the restoration). Therefore, the results of lack of significant difference between these three restorations type should be seen with caution.

The implant placement site in an area with lower bone density (low value of insertion torque) are related with lower implants' survival rate (20). In this regard, a huge amount of implant loss with machined titanium implants in type IV bone caught the attention of clinicians in the early 1990s (21). However, the modification of the titanium surface by etching resulted in an improvement of osseointegration (22). In the present study all implants received a well-recognized double etched surface treatment, which might be responsible for the lack of statistical difference in terms of implant survival rate in different implant placement sites.

In the present study the history of previous grafting did not impair the implants survival rate. A retrospective study showed that the survival rates of implants placed in grafted sites were lower than those installed in pristine bone (12). On the other hand, a recent systematic review focused on the study of survival rates of dental implants placed in sites previously grafted with autogenous and allogeneic bone blocks showed high levels for both bone graft materials (96.23 ± 5.27% and 97.66 ± 2.68%, respectively) (23). Similar results were verified by another systematic review that analyzed the outcomes of dental implants after the use of tenting for bony augmentation (24). The results of these reviews seem to corroborate our findings.

This study showed the high survival rate of implants from the same system (i.e., IntraOss implants) installed by postgraduate students in Implantology Program at São Leopoldo Mandic Dental School (Brazil), up to 52 months. This retrospective study included 742 patients who received a total of 1,164 implants showing a cumulative failure rate of 2.5%. Based on Kaplan-Meier curve analysis, with a 95% confidence interval up to 52 months of implant placement, revealed that the overall survival rate at this time was 90.5%. Clinical training in implant dentistry for graduate students contributes to the development of advanced skills in dental students. In fact, surgeons' dental/implant education may contribute to treatment outcome and implant failure rates (25). The success rate for Harvard School of Dental Medicine periodontology postgraduate students was 96.48% during the 4-year study period (26). Nonetheless, an interesting study analyzed the implant outcomes and the clinical training at Louisiana State University Health Science Center (USA), showing that the advanced group (94.2%) had the best implant outcomes followed by the intermediate group (89.38%) and beginner group (88.6%) clearly demonstrating that increased clinician training improves clinical outcomes (18). Moreover, an interesting analysis demonstrated that clinicians' age and years of experience as dentists or as specialists were not found to be predictors to early implant failure rate however, the number of implants placed during the postgraduate training was found to be significantly predicting early failure rate of implants (27). In the present study, of the 1,164 implants installed by postgraduate students in Implantology, 29 (2.5%) failed up to 52 months after implant placement. It reveals an overall survival rate of 90.5% at this time. Thus, the present data are very close to published data by several universities worldwide during the residency training.

Worth noting is that in a recent publication (25), dental implants failed after a mean time of 6.29 ± 6.75 months, reaching a survival rate of 96.9%. In our study, using the studied dental implants, we found higher survival rates of 98.8% and 98.3% up to 6 and 12 months, respectively. In our study the decrease in survival rate became more noticeable 3 years after dental implant placement. Probably, this may be attributed to a less regular attendance or nonattendance of patients to supportive periodontal/peri-implant care, which can increase the risk of dental implant failure in almost four times (28). However, one should notice that almost 85% of the placed implants in our study had up to 2 years in the oral cavity (Table 2). In effect, a limitation of the current study was the retrospective timeline of up to 52 months and the fact that the implants included had been present for variable times.

The present retrospective study showed a high survival rate, with gender, diabetes, smoking, continuous use of medication, type of implant connection system, implant position, previous bone grafting and type of prosthetic provisioning not accounting for the risk of implant failure.

## Data Availability

The datasets presented in this study can be found in the Supplementary Material.

## References

[1] Buser D Janner SFM Wittneben J-G Brägger U Ramseier CA GE S. 10-Year Survival and success rates of 511 titanium implants with a sandblasted and acid-etched surface: a retrospective study in 303 partially edentulous patients. Clin Implant Dent Relat Res. (2012) 14(6):839–51. 10.1111/j.1708-8208.2012.00456.x 22897683

[2] Jung RE Zembic A Pjetursson BE Zwahlen M Thoma DS. Systematic review of the survival rate and the incidence of biological, technical, and aesthetic complications of single crowns on implants reported in longitudinal studies with a mean follow-up of 5 years. Clin Oral Implants Res. (2012) 23:2–21. https://doi.org/10.1111/j.1600-0501.2012.02547.x 23062124

[3] Zhang L Lyu C Shang Z Niu A Liang X. Quality of life of implant-supported overdenture and conventional complete denture in restoring the edentulous mandible: a systematic review. Implant Dent. (2017) 26(6):945–50. 10.1097/ID.0000000000000668 29189390

[4] van Velzen FJ Ofec R Schulten EA Ten Bruggenkate CM. 10-year survival rate and the incidence of peri-implant disease of 374 titanium dental implants with a SLA surface: a prospective cohort study in 177 fully and partially edentulous patients. Clin Oral Implants Res. (2015) 26(10):1121–8. 10.1111/clr.12499 25370914

[5] Moraschini V Poubel LA Ferreira VF Barboza Edos S. Evaluation of survival and success rates of dental implants reported in longitudinal studies with a follow-up period of at least 10 years: a systematic review. Int J Oral Maxillofac Surg. (2015) 44(3):377–88. 10.1016/j.ijom.2014.10.023 25467739

[6] Meijer HJA Boven C Delli K Raghoebar GM. Is there an effect of crown-to-implant ratio on implant treatment outcomes? A systematic review. Clin Oral Implants Res. (2018) 29:243–52. 10.1111/clr.13338 30306696 PMC6221159

[7] Adler L Buhlin K Jansson L. Survival and complications: a 9- to 15-year retrospective follow-up of dental implant therapy. J Oral Rehabil. (2020) 47:67–77. 10.1111/joor.12866 31359446

[8] Sriram K Duraisamy R Mp SK. Survival rates of implants placed by undergraduate students: a retrospective study. J Long Term Eff Med Implants. (2020) 30(3):173–8. 10.1615/JLongTermEffMedImplants.2020035941 33463964

[9] Naito M Lung K Linke B. Retrospective analysis of the survival of dental implants placed by dental students: a 10-year chart review. J Can Dent Assoc. (2020) 86:k11. PMID: 33326369

[10] Derks J Schaller D Håkansson J Wennström JL Tomasi C Berglundh T. Effectiveness of implant therapy analyzed in a Swedish population: prevalence of peri-implantitis. J Dent Res. (2016) 95(1):43–9. 10.1177/0022034515608832 26701919

[11] French D Grandin HM Ofec R. Retrospective cohort study of 4,591 dental implants: analysis of risk indicators for bone loss and prevalence of peri-implant mucositis and peri-implantitis. J Periodontol. (2019) 90(7):691–700. 10.1002/JPER.18-0236 30644101 PMC6849729

[12] Wu X Chen S Ji W Shi B. The risk factors of early implant failure: a retrospective study of 6113 implants. Clin Implant Dent Relat Res. (2021 Jun) 23(3):280–8. 10.1111/cid.12992 33724690

[13] Naseri R Yaghini J Feizi A. Levels of smoking and dental implants failure: a systematic review and meta-analysis. J Clin Periodontol. (2020) 47(4):518–28. 10.1111/jcpe.13257 31955453

[14] Cohen A Horton ES. Progress in the treatment of type 2 diabetes: new pharmacologic approaches to improve glycemic control. Curr Med Res Opin. (2007) 23:905–17. 10.1185/030079907X182068 17407648

[15] Al Ansari Y Shahwan H Chrcanovic BR. Diabetes Mellitus and dental implants: a systematic review and meta-analysis. Materials (Basel). (2022) 15(9):3227. 10.3390/ma15093227 35591561 PMC9105616

[16] Patel V Sadiq MS Najeeb S Khurshid Z Zafar MS Heboyan A. Effects of metformin on the bioactivity and osseointegration of dental implants: a systematic review. J Taibah Univ Med Sci. (2022) 18(1):196–206. 10.1016/j.jtumed.2022.07.003 36398019 PMC9643507

[17] Lázaro-Abdulkarim A Lazaro D Salomó-Coll O Hernandez-Alfaro F Satorres M Gargallo-Albiol J. Failure of dental implants and associated risk factors in a university setting. Int J Oral Maxillofac Implants. (2022) 37(3):455–63. 10.11607/jomi.9204 35727235

[18] Sonkar J Maney P Yu Q Palaiologou A. Retrospective study to identify associations between clinician training and dental implant outcome and to compare the use of MATLAB with SAS. Int J Implant Dent. (2019) 5(1):28. 10.1186/s40729-019-0182-6 31396724 PMC6687780

[19] Kim YM Lee JB Um HS Chang BS Lee JK. Long-term effect of implant-abutment connection type on marginal bone loss and survival of dental implants. J Periodontal Implant Sci. (2022) 52(6):496–508. 10.5051/jpis.2200960048 36468468 PMC9807847

[20] Ottoni JM Oliveira ZF Mansini R Cabral AM. Correlation between placement torque and survival of single-tooth implants. Int J Oral Maxillofac Implants. (2005) 20(5):769–76. PMID: 16274152

[21] Jaffin RA Berman CL. The excessive loss of branemark fixtures in type IV bone: a 5-year analysis. J Periodontol. (1991) 62(1):2–4. 10.1902/jop.1991.62.1.2 2002427

[22] Jemat A Ghazali MJ Razali M Otsuka Y. Surface modifications and their effects on Titanium dental implants. Biomed Res Int. (2015) 2015:791725. 10.1155/2015/791725 26436097 PMC4575991

[23] Donkiewicz P Benz K Kloss-Brandstätter A Jackowski J. Survival rates of dental implants in autogenous and allogeneic bone blocks: a systematic review. Medicina (Kaunas). (2021) 57(12):1388. 10.3390/medicina57121388 34946333 PMC8705565

[24] Pourdanesh F Esmaeelinejad M Aghdashi F. Clinical outcomes of dental implants after use of tenting for bony augmentation: a systematic review. Br J Oral Maxillofac Surg. (2017) 55(10):999–1007. 10.1016/j.bjoms.2017.10.015 29174105

[25] Chatzopoulos GS Wolff LF. Dental implant failure and factors associated with treatment outcome: a retrospective study. J Stomatol Oral Maxillofac Surg. (2023) 124:S2468-7855(22)00328-7. 10.1016/j.jormas.2022.10.013 36280552

[26] Zupnik J Kim SW Ravens D Karimbux N Guze K. Factors associated with dental implant survival: a 4-year retrospective analysis. J Periodontol. (2011) 82(10):1390–5. 10.1902/jop.2011.100685 21417587

[27] Mordechai F Tali C Jonathan M Ori P Yaron B Ram S The effect of type of specialty (periodontology/oral surgery) on early implant failure: a retrospective “Big-Data” study from a nation-wide dental chain in Israel. Clin Oral Investig. (2022) 26:6159–63. 10.1007/s00784-022-04565-z 35759088 PMC9525354

[28] Carra MC Blanc-Sylvestre N Courtet A Bouchard P. Primordial and primary prevention of peri-implant diseases: a systematic review and meta-analysis. J Clin Periodontol. (2023). 10.1111/jcpe.13790. [Epub ahead of print] 36807599

